# Supplementary material for: Extracellular Release of Mitochondrial DNA: Triggered by Cigarette Smoke and Detected in COPD
Source: Cells. 2022 Jan 22;11(3):369. doi: 10.3390/cells11030369 (PMC8834490; doi:10.3390/cells11030369)
Supplement: Supplementary file 1 [file cells-11-00369-s001.zip › Giordano_SupplMater_Proofread-FINAL.pdf]

Supplementary Materials

Extracellular Release of Mitochondrial DNA: Triggered by Cigarette Smoke and Detected in COPD

Luca Giordano, Alyssa D. Gregory, Mireia Pérez Verdaguer, Sarah A. Ware, Hayley Harvey, Evan DeVallance, Tomasz Brzoska, Prithu Sundd, Yingze Zhang, Frank C. Sciurba, Steven D. Shapiro, Brett A. Kaufman

Supplementary Methods

Mean Chord Length (MCL) Measurement

The mean alveolar chord length was measured on paraffin-embedded left mouse lungs. Sections were stained with modified Gill's stain, and ten randomly selected 200x fields per slide were analyzed using Scion Image software (version 4.0.2, Scion Corp., Frederick, MD). Airway and vascular structures were masked from the analysis and the images were manually thresholded [1].

Cell Culture Conditions and CSE Exposure

BEAS-2B cells (ATCC CRL-9609, Manassas, VA) were maintained in DMEM/F12 (Gibco, Cat#11320-033) supplemented with 5% FBS (Gibco, Cat#10437-028), 100 U/ml penicillin, and 100 μg/ml streptomycin (HyClone, Logan, UT, USA, Cat#SC30010). Trypsin (Thermo Fisher Scientific, Cat#25200056) and a hemacytometer (Hausser Scientific, Horsham, PA, USA, Cat#3200) were used to detach and count cells, respectively. For the experiments, 1 x 10^5 cells were plated in a 6-well plate (Genesee Scientific, San Diego, CA, Cat#25105) and cultured overnight, then washed with PBS (Gibco, Cat#10010023) and incubated for 24 hours (h) in 2 ml of fresh medium containing cigarette smoke extract (CSE) at the concentrations indicated in the figures.

For the experiments with synthetic DNA, naked polydA:dT (double-stranded B DNA, Invivogen, cat#tlrl-patn-1) was incubated with LyoVec (Invivogen cat#lyec-1) as indicated by the manufacturer's instruction. Cells were transfected with 1 or 2 μg/ml of polydA:dT for 12 h, or treated with 1 or 2 μg/ml polydA:dT without transfectant.

All the experiments in which cells were incubated with CSE (including the unexposed cells, control) or with synthetic DNA were performed in a growth medium without FBS and antibiotics. FBS was not included in the media to avoid contaminations of extracellular vesicles (EVs) contamination [2,3]. Living cells were washed with PBS, detached with 0.025% trypsin for two minutes (min) at 37˚C, and collected by centrifugation at 1,000 x *g* for five min. The supernatant was removed and the cell pellet was resuspended in cold PBS, transferred into a new tube, centrifuged at 1,000 x *g* for five min at 4˚C, and frozen at -80˚C.

Extracellular Vesicle Isolation and Characterization

BEAS-2B cells (1 x 10^5/well) were seeded in 6-well plates and treated the following day with CSE for 24 h. One ml of growth medium was collected, centrifuged at 2,000 x *g* for 30 min to remove the cell debris, then frozen at -80˚C. The supernatant was used to isolate EVs by the Total Exosome Isolation kit (Invitrogen, Life Technologies, Carlsbad, CA, cat#4478359), as described by the manufacturer. The size and concentration of nanoparticles (*i.e.*, nanoparticle tracking analysis) were measured on fresh isolated EVs using a NanoSight NS300 instrument (NanoSight, Malvern Instruments, Malvern, UK). Samples were diluted in PBS to a final volume of 500 μl, then injected into the NS300 unit. Shutter and gain were consistent for the experimental series. A video of 60 seconds (s) was recorded three times for each sample, and then analyzed with the Nanoparticle Tracking Analysis (NTA) 2.0 Analytical software as described elsewhere [4]. Size and concentration reports were obtained for each data set.

Cell Free-DNA Extraction and Quantification

Cell-free DNA (cf-DNA) was extracted by ethanol precipitation as described previously with minor modifications [5]. Briefly, 150 μl of human plasma, or 100 μl of mouse serum, cell debris, EVs, and cell pellets, were thawed and resuspended in proteinase K buffer (100 mM Tris-HCl pH 8.5, 5 mM EDTA, 0.2% SDS, 200 mM NaCl), supplemented with 0.01% β-mercaptoethanol and 0.6 mg/ml proteinase K (Fisher Scientific, cat# 97062-238) in a final volume of 500 μl, and digested overnight at 55°C. After the addition of NaCl (to the final concentration of 1.27 M) and centrifugation at 28,000 x *g* for 15 min at 4°C to remove the protein pellet, 800 μl of 100% ethanol and 1 μl GlycoBlue coprecipitant (Thermo Fisher

Scientific, cat#AM9516) were added to the supernatant, and nucleic acids were precipitated by centrifugation at 28,000 x *g* for 15 min at 4°C. The pellet was washed with 70% ethanol, air-dried, and resuspended in DNase-free water. DNA was treated with 0.4 mg/ml RNase A (Sigma-Aldrich, cat#12091021) for 2 h at 37°C to degrade RNA, then stored at -20°C.

Cell free-mitochondrial DNA (cf-mtDNA) and cell free-nuclear DNA (cf-nDNA) were quantified by duplex qPCR using TaqMan chemistry. Primer/probe ratios of 1:1 nmole (i.e., primer-limited) for mtDNA assays and 3:1 nmoles (i.e., not primer-limited) for nDNA assays were used, designed on the mitochondrial NADH-ubiquinone oxidoreductase core subunit 1 (MT-ND1) and nuclear-encoded β2-microglobulin (B2M) for human samples [6], and a MT-ND1 and nuclear-encoded histone deacetylase 1 (HDAC1) probe set for the mouse serum. Primer set and probe sequences are described in Table S3. A final volume reaction of 10 μL containing 4 ng of template DNA and 2X Luna Universal qPCR Master Mix (New England Biolabs, Ipswich, ME, USA, cat#M3004) or 2X TaqMan Fast Advanced Master Mix (Thermo Fisher Scientific, cat#4444965) were used. Thermocycling conditions (for both, MT-ND1/B2M and MT-ND1/HDAC1) were performed in a 96-well StepOnePlus™ Real-Time PCR System (Applied Biosystems, Thermo Fisher Scientific, cat#4376600) as described: an initial denaturation step at 95°C for 20 s, followed by 40 cycles each consisting of a denaturation step at 95°C for 1 s, an annealing step at 63°C for 20 s, and an elongation step at 60°C for 20 s. Serial dilutions of DNA were used to ensure assay linearity, and to calculate the relative abundance of individual samples. Absolute copies/µl of DNA were determined by interpolation of the standard curve analyzed by singleplex Digital PCR using the QuantStudio 3D Digital PCR System (Applied Biosystem, Thermo Fisher Scientific) for each primer set. Digital PCR reactions were performed in duplicate (two chips per primer).

Cytotoxicity Assay and β-Galactosidase Staining

Cytotoxicity screening was conducted by cell density determination using a sulforhodamine B (SRB) assay to measure total cellular protein content as described previously [7,8] with some modifications. Briefly, 7 x 10^4 cells were plated in a 24-well plate overnight. Then, cells were washed with PBS and incubated for 24 h with CSE. The cell monolayer was fixed with 10% cold trichloroacetic acid (Sigma-Aldrich, St. Louis, MO, USA, cat#T6399) at 4ºC for 1 hour (h), washed with PBS and dried at 37ºC for 90 min in a heater (Isotemp, Thermo Fisher Scientific). Cells were stained with 0.4% SRB solution (Sigma-Aldrich, cat#S1402) for 20 min at room temperature (RT) on a shaker. Non-specific SRB staining was removed by five washes with 1% acetic acid, and the cell monolayer was dried at 37ºC for 90 min in the heater. The protein-bound dye was dissolved by adding 10 mM Tris (pH 10.5) to the well followed by gentle shaking for 10 min. Two-hundred µl of the dissolved solution was transferred to a 96-well plate and the absorbance was read in triplicate at 510 nm at 25°C using a Synergy H1 (BioTek) microplate reader.

β-galactosidase (β-gal) staining was performed using the Senescence β-Galactosidase Staining Kit (Cell Signaling Technology, MA, USA, cat#9860), following the manufacturer's instructions. Briefly, 1 x 10^5 cells/well were seeded in a 6-well plate overnight. Cells were treated with CSE for 24 h, followed by fixation for 15 min, rinsed with PBS, and incubated with the β-gal staining solution at 37°C overnight. After rinsing with PBS, the crystals were solubilized in 50% DMSO and images were taken by an Axiovert 40 CFL microscope (Carl Zeiss, White Plains, NY, USA) using an AxioCamMRC5 camera and AxioVision LE 4.8 software. β-gal positive cells were quantified using the cell counter plugin by ImageJ [9]. The ratio of β-gal-positive cells to the total number of cells per single image was expressed as a percentage. Images were adjusted for brightness and contrast, and resized and annotated for presentation without any further manipulation.

Mitochondrial Membrane Potential and Superoxide Production Measurements

Mitochondrial membrane potential and superoxide production were measured by Tetramethylrhodamine ethyl ester (TMRE; Abcam, Cambridge, UK, cat#ab113852, Abcam)

and MitoSOX Red (Thermo Fisher Scientific, cat#M3600) probes, respectively. A Sinergy H1 microplate reader (BioTek) was used to measure fluorescence, set at 549 nm λ_{ex} and 575 nm λ_{em} for TMRE, and 510 nm λ_{ex} and 580 nm λ_{em} for MitoSOX. Briefly, 7 x 10^5 cells were seeded in a 10 cm dish. After 24 h, the cells were washed with PBS and incubated with increasing concentrations of CSE for 3 or 6 h, as indicated in the figure legend. Then, the cells were washed with PBS, detached with trypsin, and centrifuged at 1,000 x *g* for 5 min.

To measure the mitochondrial membrane potential, cells were resuspended in fresh media containing CSE supplemented with 250 nM TMRE, and incubated for 25 min at 37°C. Next, cells were centrifuged at 1,000 x *g* for 5 min and resuspended in 1 ml of 0.02% bovine serum albumin (BSA; Sigma-Aldrich, cat#A9418) in 1X PBS. Fluorescence was measured using 1.4 x 10^4 cells/well in a 96-well black plate (Greiner Bio-One, Monroe, NC, USA, cat#655076). Cells incubated without TMRE or with 20 µM carbonyl cyanide p-trifluoro-methoxyphenyl hydrazone (FCCP) were used as negative and positive controls, respectively.

To quantify superoxide production, the fluorescence variation (ΔRFU) during a time course of 10 min was measured in 1.4 x 10^5 cells/well using a 96-well black plate (Greiner Bio-One, cat#655076) in cells previously resuspended in HBSS (Gibco, 14175) and probed with 5 µM MitoSOX. Cells pretreated with 100 µM MitoTEMPO (Enzo Life Sciences, Farmingdale, NY, USA, cat#ALX-430-150-M005), or incubated with 50 µM Antimycin A (Sigma-Aldrich, A8674) were used as negative and positive controls, respectively.

Cell Cycle Analysis

Cell cycle arrest was quantified by detecting the incorporation of 5-ethynyl-2'-deoxyuridine (EdU) during *de novo* DNA synthesis using the Click-iT EdU Alexa Fluor 488 Flow Cytometry assay Kit (Thermo Fisher Scientific, cat#C10425). Briefly, cells were co-incubated with 2 µM EdU the last 2 h of CSE exposure, then they were collected and processed following the manufacturer's instructions, and analyzed by an LSR II flow cytometer (BD Biosciences, San Jose, CA, USA). EdU-free cells were used as a negative control.

Immunocytochemistry

1 x 10^5 cells were plated in a 6-well plate and cultured overnight on a 35 mm glass coverslip (MatTEK Corporation, Ashland, MA, USA, cat#P35GCOL-1.5-10-C). Then they were incubated with CSE for 24 h, washed with PBS, fixed with 4% paraformaldehyde (Electron Microscopy Sciences, Hatfield, PA, USA, cat#15719-S) for 20 min at RT, washed again with 0.3 M glycine-PBS (3 times, 5 min each), permeabilized with 0.3 M glycine-0.1% Triton-X100 for 15 min at RT, and blocked for 30 min with 0.3 M glycine 0.05% Triton-X100, 0.5% BSA (blocking solution). Cells were stained with primary antibodies diluted in blocking solution overnight at 4°C as follows: rabbit anti-53BP1, (1:250, Abcam, cat#175933), mouse anti-α-tubulin (1:1000, Sigma Aldrich, cat#T6074), and anti-TOMM20 (1:250, Thermo Fisher Scientific, cat#PA5-52843). After rinsing with PBS (3 times, 5 min each), cells were probed for 1 h, at RT temperature with the following secondary antibodies: Alexa Fluor 647 goat anti-rabbit (1:500, Thermo Fisher Scientific, cat#A21245) and Alexa Fluor 488 goat anti-mouse (1:500, Thermo Fisher Scientific, cat#A2817) diluted in blocking solution, followed by a wash in PBS (3 times, for 5 min). Nuclei were stained using 2 µg/ml Hoechst (bis-benzimide H33258, Sigma Aldrich, cat#B2883) for 20 min at RT. Coverslips were mounted by using Gelvatol medium on a microscope slide (Thermo Fisher Scientific, cat#1255015).

Spinning Disk Confocal Microscopy and Image Analysis

All images were acquired using a spinning-disk Marianas system, based on a Zeiss Axio Observer Z1 inverted fluorescence microscope equipped with a 63X Plan Apo PH NA 1.4 oil immersion objective, Evolve EM-CCD camera, piezo stage controller, spherical aberration correction module and lasers (405, 488, 515, 561 and 640 nm), all of which were controlled by SlideBook6 software (Intelligent Imaging Innovation, Denver, CO). A z-stack of fifteen 2D-confocal images were acquired in 400 nm steps. Image acquisition settings were identical for

all variants in each experiment, using the 405 nm laser (for Hoechst), 488 nm laser (for Tubulin), and 640 nm laser (for 53BP1 and TOMM20).

To count the 53BP1 foci, 3D images were deconvolved using the constrained iterative algorithm of SlideBook6. A segment mask was then generated to select 53BP1 puncta detected through the 640 nm channel (53BP1 mask), and objects smaller than 5 voxels were eliminated. Another segment mask was generated with the minimum threshold in the 405 nm channel to include total Hoechst fluorescence (Hoechst mask), and objects smaller than 100 voxels were eliminated. For both masks, identical threshold parameters were used for all experimental variables. The number of objects in 53BP1 mask was divided by the number of objects in the Hoechst mask and reported as the number of foci/nucleus. The volume of each object of the 53BP1 mask was also calculated. Six-eight images per condition containing multiple cells were analyzed for each independent experiment.

Mitochondrial morphology analysis was carried out using maximum projection of 3D images, after background subtraction. A segment mask was created for the 640 nm channel to select the TOMM20 signal (TOMM20 mask), and objects smaller than 5 voxels were eliminated from the mask. Another segment mask was generated with the minimum threshold in the 405 nm channel to include total Hoechst fluorescence (Hoechst mask), and objects smaller than 100 voxels were eliminated. Incomplete or intertwined cells were manually removed from both masks. The number of objects (TOMM20-positive mitochondria), the total area, and the total perimeter of the TOMM20 mask were divided by the number of objects of the Hoechst mask to calculate the number of mitochondria, mitochondrial area, and perimeter per cell. For the mitochondrial morphological analysis, the area, perimeter, aspect ratio (major axis/minor axis of the ellipse equivalent to the object), and form factor ($perimeter^2/4\pi \cdot area$; inverse of sphericity) were calculated for each object of the TOMM20 mask as previously described [10]. Six-eight images per condition containing multiple cells were analyzed for each independent experiment.

Long-range PCR of the Mitochondrial Genome

Long-range PCR of mtDNA was performed as previously described [11] with minor modifications. Briefly, DNA extracted from human plasma (2.5 ng) or EVs (3 ng) was amplified using 0.5 µM primers and LongAmp Hot Start Taq 2X Master Mix (New England BioLabs, cat#M0533S) in a final volume of 50 µl, by a ProFlex PCR System Thermocycler (Applied Biosystems). Thermocycling conditions were set as follows: polymerase activation (94°C for 2 min), then 30 cycles, each consisting of denaturation (94°C for 15 seconds), annealing and elongation (62°C for 12 min), with a final extension stage (72°C for 10 min). PCR products were run on a 0.7% agarose gel. Primers used for this assay were previously described [11,12] and listed in Table S4.

RNA Extraction and RT-qPCR

RNA was extracted from 1.5 x 10^5 cells using the RNeasy Mini Kit (Qiagen, Valencia, CA, USA, cat#74104) and treated with DNase I to remove DNA contamination using the DNA-free Kit DNase Treatment and Removal Reagents (Life Technologies, cat#AM1906) following the manufacturer's instructions. RNA was quantified by NanoDrop (Thermo Fisher Scientific, NanoDrop One^C) and cDNA was synthesized using 1 µg of RNA by High-Capacity cDNA Reverse Transcription Kit (Applied Biosystems, cat#4368813) in a ProFlex PCR System Thermocycler (Applied Biosystems). Real-time qPCR was carried out using 2 µl of cDNA as template (coming from the RT-qPCR diluted 1:10 or 1:100), 0.4 µM primers, and Power Sybr Green PCR Master Mix (Applied Biosystems, cat#4367659) in a final volume of 10 µl, using a 96-well StepOnePlus™ or a 384-well QuantStudio5 Real-Time PCR System (Applied Biosystems). Primer sets were designed using the Primer Blast software (https://www.ncbi.nlm.nih.gov/tools/primer-blast) and are reported in Table S5, or were previously described [13]. Quantitative PCR was run with an initial UDG activation at 50°C for 2 min, denaturation at 95°C for 10 min, followed by 40 cycles of denaturation (95°C,

15 seconds) and annealing and synthesis (59°C, 1 min). Fold change was calculated by the $2^{-\Delta\Delta Ct}$ method using β-Glucuronidase (GUSB) as a housekeeping gene.

Protein Extraction, SDS-PAGE, and Western blotting

Snap-frozen human and mouse lung tissues were mechanically homogenized by a pestle in a cold mortar submerged in liquid nitrogen vapor. Twenty-five mg of powder were dissolved in modified RIPA buffer ((150 mM NaCl, 50 mM Tris-HCl, pH 7.4, 0.25% sodium deoxycholate, 1 mM EDTA, 1% NP-40, 1% complete protease inhibitor (Thermo Fisher Scientific, cat#36798), and 1% phosphatase inhibitor (VWR, G-Biosciences, St. Louis, MO, USA, cat#95029-242)) on a rotator at 4°C for 30 min. Cell pellets were also lysed in RIPA buffer on ice for 30 min. Lysates were centrifuged at 20,000 x *g* for 10 min at 4°C, and the supernatant was transferred to pre-chilled tubes for protein quantification using the Micro BCA Protein Assay (Life Technologies, cat#23235). Ten or fifteen µg of protein were separated on a 4-12% bis-tris polyacrylamide gel (Thermo Fisher Scientific, cat#NW04122), transferred to a nitrocellulose membrane using an iBlot gel transfer device (Invitrogen, Life Technologies), blocked with 0.25X Intercept Blocking buffer (Li-COR Biosciences, Lincoln, NE, USA, cat#927-40000), and immunoblotted overnight at 4°C using antibodies against the following proteins: GAPDH (1:2000, Cell Signaling Technology, cat#2118), MFN1 (1:2000, Cell Signaling Technology, cat#14739), MFN2 (1:2000, Cell Signaling, cat#9482), MLKL (1:1000, Cell Signaling Technology, cat#14993), OPA1 (1:1000, Cell Signaling Technology, cat#80471), p21 (1:1000, Cell Signaling Technology, cat#2947), p62 (1:1000, Sigma Aldrich, cat#PA0067), RIP1 (1:4000, Cell Signaling Technology, cat#3493), RIP3 (1:2000, Abcam, cat#56164), TFAM (1:2500, PhosphoSolutions, Aurora, CO, USA, cat#1999), Ubiquitin (1:1000, Cell Signaling Technology, cat#3933), and β-actin (1:3000, Santa Cruz Biotechnology, Dallas, TX, USA, cat#sc47778) diluted in 0.25X Intercept Blocking buffer with 0.02% Tween 20. The membrane was washed (3 times, 10 min each) with TBST (TBS, 0.001% Tween 20) and incubated for 1 h at RT with the following secondary antibodies: goat anti-mouse Alexa Fluor 680 (1:10000, Thermo Fisher Scientific, cat#A21058) and goat anti-rabbit Alexa Fluor 800 (1:10000, Thermo Fisher Scientific, cat#A32735). The membrane was washed again with TBST (3 times, 10 min each), and fluorescence was detected on a ChemiDOC MP Imaging System (Bio-Rad Laboratories, Hercules, CA, USA). After detection, the membrane was incubated with stripping solution (Li-COR Biosciences, cat#92840030) for 25 min at RT, washed with 1X TBS (2 times, 10 min each), and re-probed overnight.

Carbonylated proteins were detected using the Oxyblot Protein Oxidation Detection Kit (Millipore, Sigma-Aldrich, cat#S7150) following the manufacturer's instructions. Briefly, proteins were extracted as described above, except for the addition of 5% β-mercaptoethanol in the RIPA buffer to prevent oxidation. Equal amounts of protein (8 µg) were mixed with 5% SDS, then the carbonyl groups in the protein side chains were derived by incubating with 2,4-dinitrophenylhydrazine (DNPH) for 15 min at 30°C to generate 2,4-dinitrophenylhydrazone (DNP hydrazone). After blocking the reaction with neutralization solution for 5 min, protein samples were run on a 4-12% SDS-polyacrylamide gradient gel followed by western blotting that was performed as described above, except that the blocking agent was 3% BSA in PBS 1X, 0.1% Tween 20 (PBST). The membrane was incubated overnight with an antibody against DNP-hydrazone (included in the kit, diluted 1:400 with 1% BSA-PBST). After washing with PBST (3 times, 10 min each), the membrane was incubated for 1 h with the secondary antibody (included in the kit, diluted 1:1000 in 3% BSA PBST), and washed again with PBST (3 times, 10 min each). The immunoreactivity was detected using the chemiluminescent substrate Pierce ECL Plus (Thermo Fisher Scientific, cat#32132) on a ChemiDOC MP Imaging System (Bio-rad Laboratories). After detection of carbonylated proteins, the membrane was stripped as described above and re-probed overnight with an anti-GAPDH antibody (rabbit, 1:2000, Cell Signaling Technologies, Cat#2118S) in 1% BSA-PBST. After washing with PBST (3 times, 10 min each), the membrane was incubated with the secondary antibody goat anti-rabbit (1:12500, Santa Cruz Biotechnology cat#sc-2004) diluted in 1% BSA PBST at RT for 1 h, followed by

washes with PBST (3 times, 10 min each). The GAPDH signal (previously described) was detected and used to normalize the total carbonylated proteins.

Illustration

The schematic illustration in Figure 8 was created using BioRender.com.

Supplementary Tables

Table S1. Clinical and Demographic Characteristics of the Human Plasma Study Cohort

	**Former Smoker**	**Former Smoker COPD**	**P-value**
**N**	14	20	
**Age (years)**	63.5 ± 6.5	59.9 ± 5.1	0.006
**Gender (% Female)**	57.10%	75.00%	0.2874
**Packs per year**	48.6 ± 23.8	49.3 ± 36.1	0.9556
**FEV_1% Predicted**	99.5% ± 10.2%	23.0% ± 3.4%	<0.0001
**FEV_1/FVC**	0.793 ± 0.03	0.318 ± 0.06	<0.0001
**DLCO% Predicted**	91.6% ± 12.7%	29.7% ± 9.0%	<0.0001
**LAA%**	0.004 ± 0.002	0.277 ± 0.121	<0.0001
**Hist 15**	-884.2 ± 23.6	-964.6 ± 15.4	<0.0001

FEV_1, forced expiratory volume in 1 second; FVC, forced vital capacity; DLCO, diffusing capacity of carbon monoxide; LAA%, percentage of low-attenuation area less than −950 Hounsfield Unit on a CT scan; Hist 15, the lower 15th percentile of the Hounsfield Unit value histogram. P-values were determined by an unpaired parametric t-test.

Table S2. Gender Characteristics of the Human Lung Tissue

Sex	Donor	COPD
Male	1	6
Female	4	5
Unknown	7	1

Table S3. Primers and Probes Used for mtDNA and nDNA Quantification

	**Primers Probes**	**5′-3′ sequence**
**human MT-ND1**	F	gagcgatggtgagagctaaggt
	R	ccctaaaacccgccacatct
	Probe	5HEX/ccatcaccc/ZEN/tctacatcaccgccc/3IABKFQ
**human B2M**	F	tctctctccattcttcagtaagtcaact
	R	ccagcagagaatggaaagtcaa
	Probe	6-FAM/atgtgtctg/ZEN/ggtttcatccatccgaca/3IABKFQ
**mouse MT-ND1**	F	gagtgatagggtaggtgcaataa
	R	ccatttgcagacgccataaa
	Probe	5HEX/agaaccaat/ZEN/acgccctttaacaacctct/3IABKFG
**mouse HDAC1**	F	ccaggtactcgttagtgttctg
	R	ctttgaatactttggaccggattt
	Probe	56-FAM/acatcagcc/ZEN/cttccaacatgacca/3IABKFQ
F and R indicate forward and reverse primers, respectively.		

Table S4. Primer Sets Used for Long-range PCR of the Mitochondrial Genome

Primers		5′-3′ sequence	Amplicon	Reference
**Mito 1**	16331 F	acatagcacattacagtcaaatcccttctcgtcccc	3968 bp	Sansone P. et al., 2017
	3729 R	tgagattgtttgggctactgctcgcagtgc		
**Mito 2**	3646 F	tactcaatcctctgatcagggtgagcatcaaactc	5513 bp	
	9458 R	gcttggattaaggcgacagcgatttctaggatagt		
**Mito 3**	8753 F	tcatttttattgccacaactaacctcctcggactc	7813 bp	
	16566 R	cgtgatgtcttatttaaggggaacgtgtgggctat		
**Mito 4**	14841 F	tttcatcatgcggagatgttggatgg	8842 bp	Furda AM et al., 2014
	5999 R	tctaagcctccttattcgagccga		
**Mito 5**	14841 F	tttcatcatgcggagatgttggatgg	221 bp	
	14620 R	ccccacaaaccccattactaaaccca		
F and R indicate forward and reverse primers, respectively; bp, base pair.				

Table S5. Primer Sets Used for Gene Expression Quantification

NM	Gene	Primer	5′-3′ sequence
NM_138441.3	cGAS	F	aggcctgcgcattcaaaact
		R	tgtgagagaaggatagccgcc
NM_005223	DNase I	F	ccagacacctatcactacgtgg
		R	ctctcggttgaaggtgtcgttc
NM_001375	DNase II	F	gtcaagggccaccacgttag
		R	gccaaccagccggagtacag
NM_033629	DNase III	F	agagtgtgcagccgagtcac
		R	ccagtggcctccatgtcgaa
NM_000181	GUSB	F	ctgtcaccaagagccagttcct
		R	ggttgaagtccttcaccagcag
NM_001562.4	IL-18	F	gatagccagcctagaggtatgg
		R	ccttgatgttatcaggaggattca
NM_004895.5	NLRP3	F	ggactgaagcacctgttgtgca
		R	tcctgagtctcccaaggcattc
NM_138554.5	TLR-4	F	ccctgaggcatttaggcagcta
		R	accctgaggcatttaggcagcta
F and R indicate forward and reverse primers, respectively.			

Supplementary Figures

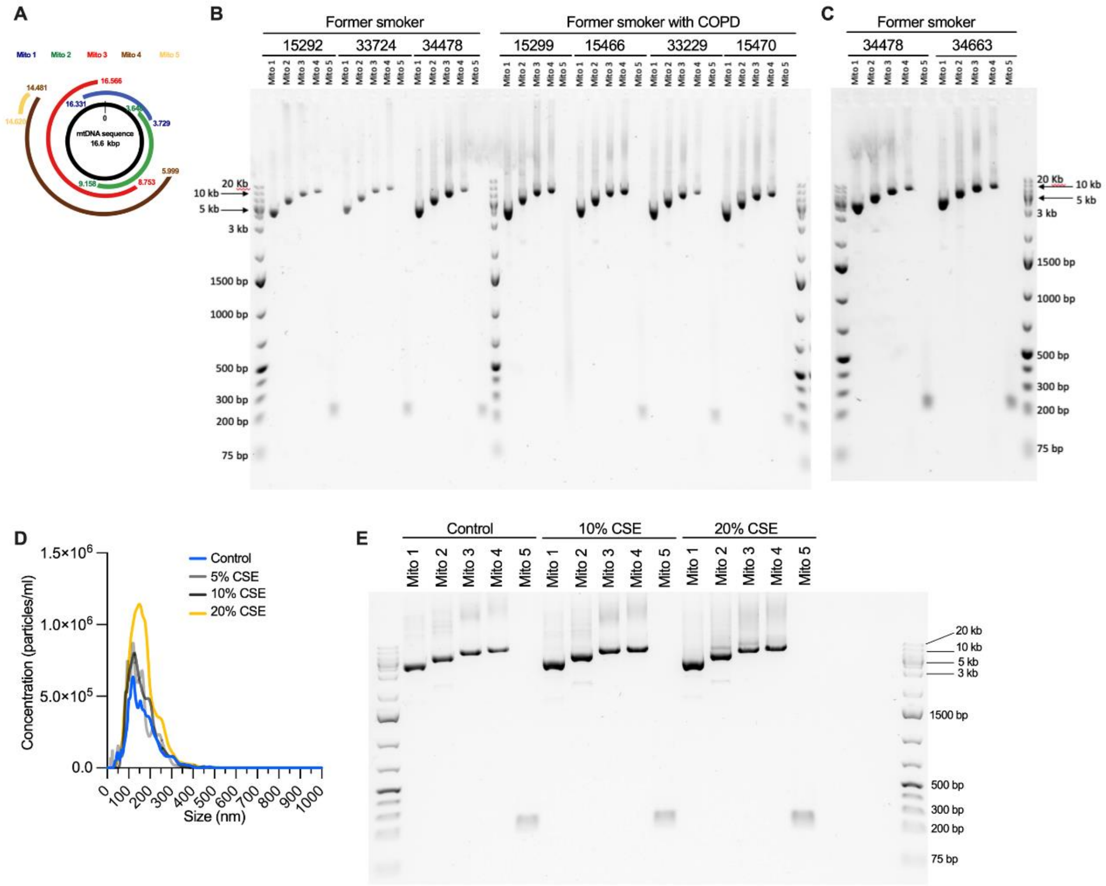

Figure S1. Human plasma of former smokers with and without COPD, and EVs released from BEAS-2B cells unexposed and exposed to CSE contain overlapping fragments of the mitochondrial genome. (**A**) Schematic and (**B-C**) representative images of agarose gel electrophoresis (AGE) of long-range PCR products amplified by five overlapping primer sets spanning the entire mitochondrial genome (Mito1-Mito5). The DNA template was extracted from a random subgroup of former smokers without COPD (*n* = 4; ID: 15292, 33724, 34478, 34663) and former smokers with COPD (*n* = 4; ID15299, 15466, 33229, 15470). (**D**) Distribution of the EVs released into the extracellular milieu by BEAS-2B cells exposed to increasing doses of CSE. (**E**) Representative image of AGE of long-range PCR products (Mito1-Mito5) performed using DNA extracted from EVs isolated from BEAS-2B cells unexposed or exposed to 10% and 20% CSE for 24 h (*n* = 2 per group). (**D**) Data are reported as mean of three independent experiments (*n* = 3). CSE, cigarette smoke extract; control, CSE-unexposed cells; EVs, extracellular vesicles; PCR, polymerase chain reaction.

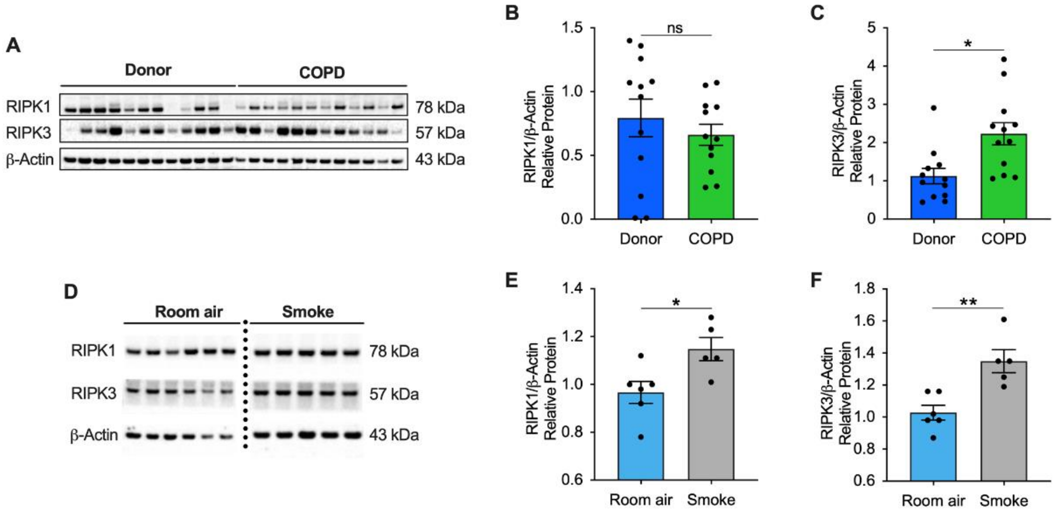

Figure S2. Necroptosis markers are dysregulated in the airways of COPD patients and in the lungs of a mouse model of emphysema induced by CS exposure. (**A,D**) Representative western blots showing RIP1 and RIP3 proteins and their relative densitometry (**B,C**) in human airways without signs of lung obstruction (Donor, $n = 12$) or affected by COPD (COPD, $n = 12$), and in (**E,F**) lung tissue of mice exposed to cigarette smoke ($n = 5$) or room air ($n = 6$) for six months. β-actin was used as a loading control. (D) The dotted arrow indicates the cropping of unrelated samples run on the same gel. Data are reported as mean ± SEM of several samples (n) indicated as circles. ns, no statistical significance; * $p < 0.05$, ** $p < 0.01$, were determined by an unpaired t-test analysis with Welch's correction.

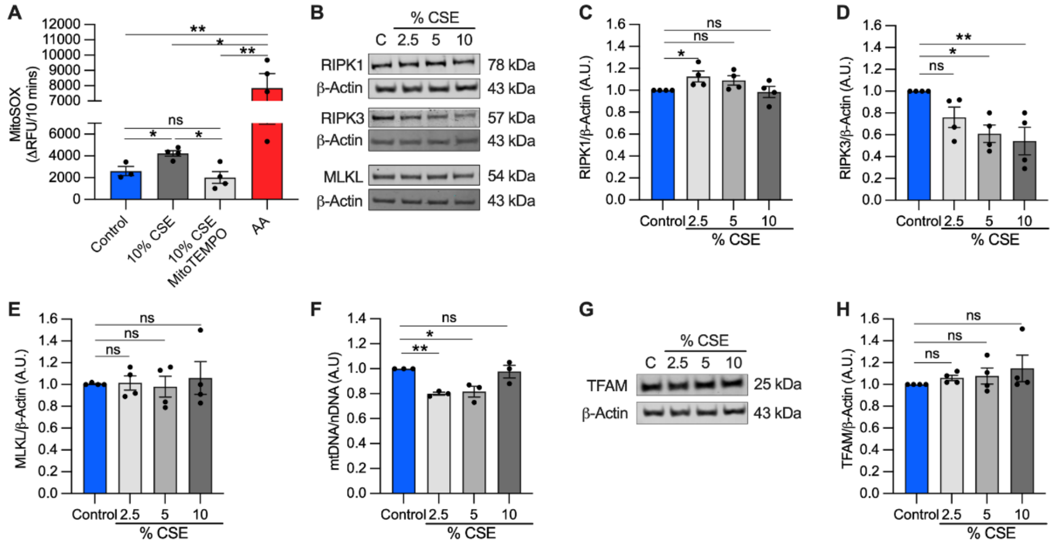

Figure S3. Exposure to a sublethal dose of CSE induces superoxide production that is neutralized by MitoTEMPO, but does not increase RIPK1, RIPK3 proteins, and the markers of mitochondrial mass (mtDNA and TFAM content). (**A**) Bar graph reporting the superoxide anion production determined by MitoSOX as the variation of the relative fluorescence unit (RFU) for 10 min from BEAS-2B cells previously exposed to CSE for 3 h, as reported in Figure 4B. Treatment groups include cells pretreated for 5 h with 100 μM MitoTEMPO or incubated with 50 μM (AA) Antimycin A, which were used as a negative and positive control, respectively. Representative western blots of (**B**) necroptosis markers (RIPK1, RIPK3, MLKL) and (**G**) TFAM in BEAS-2B cells exposed to increasing doses of CSE. Bar graphs reporting the densitometry of (**C**) RIPK1, (**D**) RIPK3, (**E**) MLKL and (**H**) TFAM proteins, and the (**F**) intracellular mitochondrial DNA content. β-actin was used as a loading control. (A,C-F,H) Data are reported as mean ± SEM of independent experiments ($n \geq 3$), indicated as circles. CSE, cigarette smoke extract; control, CSE-unexposed cells. ns, no statistical significance; * $p < 0.05$, ** $p < 0.01$ were

determined by (A) unpaired t-test with Welch's correction, (C-F,H) one-way ANOVA with multiple comparisons with Bonferroni *post hoc* test.

Supplementary Discussion

Discussion S1. Cf-DNA Detection in Mouse Models of Lung Inflammation

In C57BL/6J mice, acute exposure to CS (10 cigarettes) increased extracellular mtDNA in the BAL, as early as 30 minutes after exposure. nDNA was found 10 days later, most likely as a secondary effect of tissue necrosis [14]. Although we found similar results, our data are novel because: i) mtDNA was detected only in the BAL [14]. We detected mtDNA in the serum of mice (Figure 1E); ii) mice did not show any lung damage [14], we used chronic CS-exposure (6 months), that induces lung damage and mimics human emphysema (Figure 1C-D); iii) the study did not present a human cohort [14]. We detected increased levels of cf-mtDNA in a COPD human cohort (Figure 1A).

In the same report [14], BEAS-2B cells exposed to prolonged low-level oxidative stress induced by incubation with glucose oxidase showed moderate bioenergetic defects, mtDNA extrusion, and a proinflammatory response characterized by increased IL-1β, IL-6, and IL-8 expression. Likewise, in our study, we found increased oxidative stress (Figure 4A-D), mtDNA release by EVs (Figure 3B), and upregulated proinflammatory cytokines (Figure 6C-D) when we exposed the same cell line to CSE, a model that more accurately mimics CS exposure than glucose oxidase.

Other work, using a different paradigm of acute CS exposure (four cigarettes, three times a day for four consecutive days) to induce mouse lung inflammation, detected double-strand DNA (ds-DNA, without specifying mtDNA or nDNA) by PicoGreen fluorescent dye in the BAL of C57BL/6J mice. Importantly, the lungs of CS-exposed mice showed upregulated cGAS/STING (stimulator of interferon genes) and type I interferon (IFN I) signaling, together with neutrophil recruitment [15]. ds-DNA was also detected in the BAL of other mouse strains (BALB/cByJ, DBA/1J, C57BL/6J) except for C58/J, acutely exposed (3-5 cigarettes, five days) to CS [16].

Discussion S2. Cf-mtDNA Variations and Intracellular mtDNA Content

In cells exposed to sublethal CSE (10%) dose, the increased mtDNA in the EVs did not influence the intracellular mtDNA content and TFAM levels (Figure S3F-H), suggesting that the amount of cf-mtDNA released is insufficient to cause detectable depletion among the hundreds of mtDNA copies per cell. Furthermore, at the same sublethal dose of (10%) CSE, 35% of these cells were senescent (Figure 4H-I). Senescence has been shown to increase the mitochondrial mass [17,18]. and would be expected to offset cellular decrease of mtDNA content.

Discussion S3. Alteration of Mitochondrial Dynamics in Cells and Human Lung Tissue

Although mitochondrial fusion protein (L-OPA1, MFN1 and MFN2) were slightly decreased in BEAS-2B cells exposed to a sublethal dose of CSE, and mitochondrial fission was expected, those variations were minimal (29% for L-OPA, 20% for MFN1 and MFN2) (Figure 5I-J, L-M). We do not think they could strongly impact mitochondrial dynamics. On the contrary, the decrease of these proteins could be related to a wide degradation process induced by high oxidative stress (Figure 4B-D), as suggested by the high amount of ubiquitinated proteins (Figure 4E-F). Our results agree with a recent report that showed decreased L-OPA1 protein in several cell lines (including BEAS-2B cells) exposed to acute CSE for 4 hours [19]. Similarly, decreased MFN1 protein was detected in the mitochondrial fraction obtained from lung tissue isolated from smokers without emphysema compared to non-smokers, and in lung homogenates isolated from an area with severe emphysema compared to an area with mild emphysema in the same patient [20].

Discussion S4. Alternative Pathways That Could Induce mtDNA Release in Cells Exposed to CSE

It has been shown that IL-1β induced mtDNA release into the cytoplasm of type II alveolar epithelial cells (AECII) and macrophages in the absence of cell death [21]. Cytoplasmic mtDNA subsequently activated the innate immune response through the cGAS/STING/interferon regulatory factor 3 (IRF3) cascade [21]. Alternatively, mtDNA release may serve as a genotoxic sentinel of nDNA damage, which triggers adaptive gene expression to aid in preserving the nuclear genome [22,23]. Indeed, we showed that cells treated with a sublethal dose (10%) of CSE had increased IL-1β expression (Figure 6D), nDNA damage, and a p16-/p21-dependent cell cycle arrest (Figure 2D-F, and 4J-L). Similarly, other findings have described a p21-dependent and p53-independent cell cycle arrest in AECII from human emphysematous lungs [24].

Discussion S5. Limitations of This Study
There are four areas of potential limitations: i) While our *in vivo* data in emphysematous mice and human former smokers with COPD confirmed high levels of cf-mtDNA in the serum and plasma, our findings need to be validated in a large cohort of patients and intersected in a longitudinal study with clinical parameters to understand the potential impact on the disease. It will also be useful to investigate the presence of cf-DNA in EVs isolated from the plasma and BAL of smokers, COPD patients, and healthy control subjects. ii) CSE is a surrogate for smoke exposure in cultured cells but does not identically recapitulate CS exposure. Patient-derived cells from bronchial brushings may validate our observations. iii) while we used a representative immortalized cell line (BEAS-2B) commonly used in the field, the airways and lungs host multiple cell types (type I and II alveolar epithelial cells, endothelial, smooth muscle, and immune cells) that could be involved in DNA release or its clearance. Future experiments should investigate the role of each cell type. iv) whether extruded mtDNA acts as an inflammatory signal by binding DSRs, or whether it is involved in restoring mitochondrial bioenergetics, or is simply a marker for mitochondrial dysfunction in the lung are unresolved questions. Forthcoming studies should answer these questions by using several knockout cell and animal models.

Supplementary References

1. Radder JE, Gregory AD, Leme AS, Cho MH, Chu Y, Kelly NJ, Bakke P, Gulsvik A, Litonjua AA, Sparrow D, et al. Variable susceptibility to cigarette smoke-induced emphysema in 34 inbred strains of mice implicates abi3bp in emphysema susceptibility. *Am J Respir Cell Mol Biol* 2017;57(3):367-375.
2. Lehrich BM, Liang Y, Khosravi P, Federoff HJ, Fiandaca MS. Fetal bovine serum-derived extracellular vesicles persist within vesicle-depleted culture media. *Int J Mol Sci* 2018;19(11).
3. Lehrich BM, Liang Y, Fiandaca MS. Foetal bovine serum influence on in vitro extracellular vesicle analyses. *J Extracell Vesicles* 2021;10(3):e12061.
4. Vats R, Brzoska T, Bennewitz MF, Jimenez MA, Pradhan-Sundd T, Tutuncuoglu E, Jonassaint J, Gutierrez E, Watkins SC, Shiva S, et al. Platelet extracellular vesicles drive inflammasome-il-1β-dependent lung injury in sickle cell disease. *Am J Respir Crit Care Med* 2020;201(1):33-46.
5. Kolesar JE, Wang CY, Taguchi YV, Chou SH, Kaufman BA. Two-dimensional intact mitochondrial dna agarose electrophoresis reveals the structural complexity of the mammalian mitochondrial genome. *Nucleic Acids Res* 2013;41(4):e58.
6. Ware SA, Desai N, Lopez M, Leach D, Zhang Y, Giordano L, Nouraie M, Picard M, Kaufman BA. An automated, high-throughput methodology optimized for quantitative cell-free mitochondrial and nuclear dna isolation from plasma. *J Biol Chem* 2020;295(46):15677-15691.
7. Vichai V, Kirtikara K. Sulforhodamine b colorimetric assay for cytotoxicity screening. *Nat Protoc* 2006;1(3):1112-1116.
8. Giordano L, Deceglie S, d'Adamo P, Valentino ML, La Morgia C, Fracasso F, Roberti M, Cappellari M, Petrosillo G, Ciaravolo S, et al. Cigarette toxicity triggers leber's hereditary optic neuropathy by affecting mtdna copy number, oxidative phosphorylation and ros detoxification pathways. *Cell Death Dis* 2015;6:e2021.

9. Schindelin J, Arganda-Carreras I, Frise E, Kaynig V, Longair M, Pietzsch T, Preibisch S, Rueden C, Saalfeld S, Schmid B, et al. Fiji: An open-source platform for biological-image analysis. *Nat Methods* 2012;9(7):676-682.
10. Koopman WJ, Verkaart S, Visch HJ, van der Westhuizen FH, Murphy MP, van den Heuvel LW, Smeitink JA, Willems PH. Inhibition of complex i of the electron transport chain causes o2-. -mediated mitochondrial outgrowth. *Am J Physiol Cell Physiol* 2005;288(6):C1440-1450.
11. Sansone P, Savini C, Kurelac I, Chang Q, Amato LB, Strillacci A, Stepanova A, Iommarini L, Mastroleo C, Daly L, et al. Packaging and transfer of mitochondrial dna via exosomes regulate escape from dormancy in hormonal therapy-resistant breast cancer. *Proc Natl Acad Sci U S A* 2017;114(43):E9066-E9075.
12. Furda AM, Bess AS, Meyer JN, Van Houten B. Analysis of dna damage and repair in nuclear and mitochondrial dna of animal cells using quantitative pcr. *Methods Mol Biol* 2012;920:111-132.
13. Noren Hooten N, Evans MK. Techniques to induce and quantify cellular senescence. *J Vis Exp* 2017(123).
14. Szczesny B, Marcatti M, Ahmad A, Montalbano M, Brunyánszki A, Bibli SI, Papapetropoulos A, Szabo C. Mitochondrial dna damage and subsequent activation of z-dna binding protein 1 links oxidative stress to inflammation in epithelial cells. *Sci Rep* 2018;8(1):914.
15. Nascimento M, Gombault A, Lacerda-Queiroz N, Panek C, Savigny F, Sbeity M, Bourinet M, Le Bert M, Riteau N, Ryffel B, et al. Self-dna release and sting-dependent sensing drives inflammation to cigarette smoke in mice. *Sci Rep* 2019;9(1):14848.
16. Pouwels SD, Heijink IH, van Oosterhout AJ, Nawijn MC. A specific damp profile identifies susceptibility to smoke-induced airway inflammation. *Eur Respir J* 2014;43(4):1183-1186.
17. Dalle Pezze P, Nelson G, Otten EG, Korolchuk VI, Kirkwood TB, von Zglinicki T, Shanley DP. Dynamic modelling of pathways to cellular senescence reveals strategies for targeted interventions. *PLoS Comput Biol* 2014;10(8):e1003728.
18. Korolchuk VI, Miwa S, Carroll B, von Zglinicki T. Mitochondria in cell senescence: Is mitophagy the weakest link? *EBioMedicine* 2017;21:7-13.
19. Maremanda KP, Sundar IK, Rahman I. Role of inner mitochondrial protein opa1 in mitochondrial dysfunction by tobacco smoking and in the pathogenesis of copd. *Redox Biol* 2021;45:102055.
20. Kosmider B, Lin CR, Karim L, Tomar D, Vlasenko L, Marchetti N, Bolla S, Madesh M, Criner GJ, Bahmed K. Mitochondrial dysfunction in human primary alveolar type ii cells in emphysema. *EBioMedicine* 2019;46:305-316.
21. Aarreberg LD, Esser-Nobis K, Driscoll C, Shuvarikov A, Roby JA, Gale M. Interleukin-1β induces mtdna release to activate innate immune signaling via cgas-sting. *Mol Cell* 2019;74(4):801-815.e806.
22. Wu Z, Oeck S, West AP, Mangalhara KC, Sainz AG, Newman LE, Zhang XO, Wu L, Yan Q, Bosenberg M, et al. Mitochondrial dna stress signalling protects the nuclear genome. *Nat Metab* 2019;1(12):1209-1218.
23. Wu Z OS, West AP, Mangalhara KC, Sainz AG, Newman LE, Zhang XO, Wu L, Yan Q, Bosenberg M, Liu Y, Sulkowski PL, Tripple V, Kaech SM, Glazer PM & Shadel GS. Mitochondrial dna stress signalling protects the nuclear genome. Nature Metabolism;2019.
24. Kosmider B, Lin CR, Vlasenko L, Marchetti N, Bolla S, Criner GJ, Messier E, Reisdorph N, Powell RL, Madesh M, et al. Impaired non-homologous end joining in human primary alveolar type ii cells in emphysema. *Sci Rep* 2019;9(1):920.
